# Clinical Efficacy Comparisons Between Poly‐L‐Lactic Acid Injections and Non‐Ablative 1565‐nm Fractional Laser for Treatment of Striae Distensae—A Randomized Trial

**DOI:** 10.1111/jocd.70338

**Published:** 2025-07-15

**Authors:** Huanhuan Qu, Li Wang, Wuyan Xin, Meiheng Lu, Huan Jing, Gang Wang, Lin Gao

**Affiliations:** ^1^ Department of Dermatology, Xijing Hospital The Fourth Military Medical University Xi'an Shaanxi China

**Keywords:** 1565‐nm non‐ablative fractional laser, biostimulators, poly‐L‐lactic acid, striae distensae

## Abstract

**Background:**

Despite numerous treatment modalities for striae distensae (SD), a definitive gold standard therapy remains unidentified.

**Aims:**

This study aims to evaluate and compare the efficacy of injectable poly‐L‐lactic acid (PLLA) with a 1565‐nm non‐ablative fractional laser (NAFL) in treating SD located in the abdominal area.

**Methods:**

40 women with SD were randomly assigned to one of four treatment groups: (1) control, (2) PLLA, (3) 1565‐nm NAFL, and (4) a combination of PLLA and 1565‐nm NAFL. Participants, except those in the control group, underwent three treatment sessions at monthly intervals. Antera 3D imaging was used to collect data at baseline (T0) and 3 months post‐treatment (T4). Collagen fibers were analyzed via immunohistochemical staining, while elastic fibers were assessed using picrosirius red and Masson staining at both T0 and T4.

**Results:**

The overall efficacy scores for the PLLA, 1565‐nm NAFL, and PLLA + 1565‐nm NAFL groups were 5.70 ± 1.25, 3.60 ± 2.12, and 6.70 ± 2.21, respectively. Post‐treatment evaluations demonstrated substantial decreases in SD volume from T0 to T4, with reductions of −1.96 ± 1.53 in the PLLA group, −0.70 ± 0.67 in the 1565‐nm NAFL group, and −1.48 ± 1.35 in the PLLA + 1565‐nm NAFL group. The combined and PLLA groups exhibited significant reductions in area compared to the 1565‐nm NAFL and control groups. Histological analysis confirmed the presence of PLLA particles in the treated area without inflammatory reactions 1 month post‐final injection.

**Conclusions:**

PLLA injections are more effective than 1565‐nm NAFL in SD treatment, enhancing collagen production without inducing inflammation in treated skin.

**Trial Registration:**

Clinicaltrials.gov: NCT05827913

## 1 Introduction

Striae distensae (SD), or stretch marks, are common dermal lesions that arise due to the stretching of the dermis [1]. These dermal lesions may occur during pregnancy, adolescence, or due to obesity and certain diseases [2]. SD is often observed on the abdomen, breasts, buttocks, and thighs [3]. The prevalence of SD has been reported as varying from 11% to 88%, with the vast majority among pregnant women and adolescents. Featuring visible linear scars, SD is one of the most common cosmetic problems caused by the loss of collagen and elastin fibers in the dermis [4]. Due to its aesthetic impact, this type of atrophic scarring may cause depression and psychological problems in patients and affect their quality of life [5]. Numerous techniques have been used to stimulate the formation of collagens for the treatment of SD, including topical creams, chemical peels, microdermabrasion, pulsed dye lasers, diode lasers, ablative and non‐ablative lasers, intense pulsed light, micro‐needling, fractionated microneedle radiofrequency, dermal filler injections, and others [6]. However, the most effective therapeutic strategy for improving the appearance of SD has not yet been determined [7].

The 1565‐nm non‐ablative fractional laser (NAFL) has been shown to be a promising and effective treatment for SD, yielding improvements in the texture, volume, and color of SD with slight adverse effects [8]. In addition, many studies have proposed fillers for treating SD [9]. For example, Poly‐L‐lactic acid (PLLA)—a biocompatible, biodegradable, and bioresorbable polymer—has been safely used in many applications and medicine for more than 30 years [10]. In a study with six participants, PLLA injection for SD resulted in GAIS improvements in most subjects treated [11]. Specifically, NAFL adopts a focused approach, employing thermal energy to establish microthermal treatment zones (MTZs) within the skin. Although this precise heat application selectively damages the dermis, it preserves the epidermis, thus triggering the body's healing processes and stimulating new collagen production [12]. The polymer microspheres in PLLA dermal filler have additional biological effects that trigger collagen synthesis [13]. Since both 1565‐nm NAFL and PLLA injections render promising outcomes, we attempted to evaluate and compare the efficacy and safety of these two modalities for SD using an Antera 3D dermoscope system. In addition, dermal histopathologies were performed to assess the contents of types I and III collagens and elastic fibers.

## 2 Materials and Methods

### 2.1 Treatment Protocol

This was a single‐center, prospective, randomized, self‐controlled study. It was conducted at the Xijing Hospital, the Fourth Military Medical University, China. The study was approved by the Fourth Military Medical University Institutional Review Board for Ethics in human subject research on 14 Feb 2023 (protocol number KY20232083‐C‐1). A total of 40 female participants with SD were included in this study, characterized by white stretch marks typically associated with pregnancy. The subjects had III–IV Fitzpatrick skin types. There are four groups: control group, PLLA group, 1565‐nm NAFL group, and PLLA + 1565‐nm NAFL group. All eligible participants were randomly assigned to one of the four treatment groups using a computerized random number table. Each participant was allocated a unique identification number, and a random sequence was generated to determine group assignments. The allocation sequence was concealed in sealed, opaque envelopes, which were opened sequentially after enrollment to ensure allocation concealment. The control and experimental groups should follow identical criteria. Demographic data, age of striae, and medical history were recorded for all subjects before treatment. Topical anesthesia (2.5% lidocaine hydrochloride and 2.5% prilocaine cream, Beijing, China) was applied and left under occlusion for 1 h before the procedure. An M22‐ResurFx laser (Lumenis Inc., San Jose, California, USA) was used for NAFL treatment. The NAFL had a wavelength of 1565 nm, a selected energy of 45 mJ, and a fractional density of 200 points/cm^2^ for one pass. The endpoint reaction was erythema and wheals at the treatment site. PLLA (LÖVIselle, SinoBiom, Changchun, China) injections were given using a 1‐mL disposable sterile syringe (minimum scale 0.1 mL) and a 30G sterile injection needle. Routine disinfection was carried out before treatment. The subcutaneous and dermis layer was chosen when injecting, with the spacing between injection points of PLLA being 1 cm. The single‐point injection dosage of PLLA is 0.2 mL, with the total injection volume not exceeding 8 mL. After the PLLA was injected, fusidic acid cream (Fucidin, LEO, Denmark) was applied at the needle hole to prevent infection, and a cold compress was applied to the injection area for 15 min to reduce pain and other discomforts. In the combined treatment, the PLLA was injected immediately after 1565‐nm NAFL treatment. Otherwise, the combined treatment followed the aforementioned protocols for NAFL treatment and PLLA injection. All patients received a total of three treatments at 1‐month intervals. Figure S1 illustrates the immediate responses observed following the treatments.

### 2.2 Histopathological Examination

A 6.0‐mm biopsy punch was used to obtain specimens before treatment and 3 months after the last treatment session. This second specimen was taken 1 cm away from the first biopsy site. The samples were fixed in 4% paraformaldehyde in phosphate‐buffered saline (PBS, pH = 7.4), embedded in paraffin, and sectioned at a thickness of 4 μm. Tissue sections were deparaffinized, hydrated, and stained using H&E stain, picrosirius red stain, Masson trichrome stain, and types I and III collagen analysis. A blinded dermatopathologist compared the specimens before and after treatments and evaluated them for inflammatory infiltrations, neocollagenesis, and neoelastogenesis. The inflammatory reaction and collagen/elastic neoformation were graded on a range from 0 to 5, where 0 = no alteration and 5 = very intense alterations. Images of H&E‐ and picrosirius red–stained sections were taken at 200 × magnification using an Olympus DP74 digital camera (Olympus Optical Co., Tokyo, Japan) connected to an Olympus BX53 optical microscope (Olympus Optical Co., Tokyo, Japan). The images were analyzed using Image‐Pro‐Plus (version 6.0) image analysis software (Media Cybernetics Inc., Rockville, Maryland, USA). Changes in types I and III collagen fibers with immunohistochemical staining were assessed by analyzing their percentage density (PD) and the ratio of type I to type III collagens; elastic fiber content was assessed by analyzing PD and optical density (OD). Elastic fiber content by picrosirius red staining and Masson trichrome staining was assessed by analysis of their OD.

### 2.3 Evaluation

Follow‐up assessments were carried out at 1, 2, and 3 months after the final treatment. Objective assessments for the SD volume, area of influence, and maximum depth were performed using a 3D skin imaging device (Antera 3D CS, Miravex Limited, Dublin, Ireland). The images were taken before treatment (referred to as the time of T0), at 1 month after every treatment, and at 3 months after the last treatment (referred to as the time of T4). Standardized digital photographs (SEM; Hitachi TM3000, Hitachi, Tokyo, Japan) with identical camera settings, lighting, and patient positioning were obtained at every visit. Overall clinical efficacy score was evaluated by two independent, board‐certified dermatologists using a 4‐point grading scale (0 = no improvement, 1 = less than 25% (minimal), 2 = 25%–50% (moderate), 3 = 51%–75% (marked), 4 = more than 75% (excellent) improvement) at 6 months after the first treatment [14]. Skin elasticity, color, SD area, and degree of unevenness were evaluated. A self‐administered questionnaire with a 10‐point grading scale (0 = unsatisfactory to 10 = very satisfactory) was used to assess patients' satisfaction rates at 3 months after the last treatment. Pain scores were recorded immediately after each treatment using a 10‐point pain scale (0 = no pain to 10 = severe pain). The subjects were instructed to track their healing time, including the duration of erythema and scabbing of the treatment area; they were requested to report at every treatment visit. All adverse effects were recorded during the study. Assessments were conducted before the first treatment (T0) and 3 months after the last treatment (T4). In cases where the evaluations of the two blinded researchers disagree, a third trained, blinded researcher will be consulted to perform an independent assessment. The final outcome will be established based on the consensus of any two of the three evaluations. If all three evaluations yield differing results, the final outcome will be determined by calculating the average of their scores.

### 2.4 Statistical Analyses

SPSS 25.0 (SPSS Ltd., USA) was used for statistical analyses. For quantitative indicators, the mean, standard deviation, median, minimum, maximum, and 25% and 75% quartiles were presented. For categorical indicators, the number of examples and percentages for each category were used. ANOVA was used for between‐group comparisons. Chi‐square tests/exact probability method was used for between‐group comparisons of qualitative variables. The Kruskal‐Wallis/Wilcoxon rank‐sum tests were used for between‐group comparisons of ordered categorical data. All statistical tests were two‐sided, and *p* values less than or equal to 0.05 were considered statistically significant for the tested differences. Two‐by‐two comparisons were made using Bonferroni to adjust the level of the test standard. The statistical analysis software was SAS 9.4.

## 3 Results

### 3.1 Patient Characteristics

The 40 subjects ranged from 29 to 44 years old (36.00 ± 4.16 years). None of them had any previous SD treatment history. The mean SD duration among the subjects was 7.73 ± 3.74 years (range = 0.3–15 years). No correlation was found between the patient age and SD duration among the four groups. PLLA and PLLA +1565‐nm NAFL groups showed better outcomes than the 1565‐nm NAFL only. Table 1 shows the baseline data for each group for SD volume, area of influence, and maximum depth based on Antera 3D imaging. There were no significant differences in mean values of the SD volume, baseline SD area of influence, and baseline SD maximum depth among the control, PLLA, 1565‐nm NAFL, and PLLA +1565‐nm NAFL groups before treatment (Table 1).

**TABLE 1:** Baseline SD volume, area of influence, and maximum depth data from Antera 3D imaging.

	PLLA +1565‐nm NAFL group (*n* = 10)	PLLA group (*n* = 10)	1565‐nm NAFL group (*n* = 10)	Control group (*n* = 10)	*p*
SD volume (mm^3^)					
Mean ± s.d.	2.37 ± 2.32	3.00 ± 1.89	2.86 ± 1.60	3.11 ± 1.46	0.891
Median	1.615	2.37	2.615	2.72	
*P*25–*P*75	1.33–2.33	1.78–3.52	1.57–3.84	1.94–4.03	
Min–max	0.93–8.82	0.915–6.67	1.02–5.87	1.58–5.93	
SD area of influence (mm^3^)					
Mean ± s.d.	159.80 ± 32.87	160.90 ± 37.29	155.82 ± 31.60	158.38 ± 38.46	0.823
Median	88.92	87.86	89.83	91.85	
*P*25–*P*75	67.8–126.9	58.7–124.3	59.2–128.5	68.3–127.9	
Min–max	41.4–207.1	39.9–204.3	39.6–198.5	58.4–185.3	
SD maximum depth (mm)					
Mean ± s.d.	0.08 ± 0.09	0.07 ± 0.03	0.06 ± 0.01	0.06 ± 0.01	0.535
Median	0.052	0.054	0.062	0.064	
*P*25–*P*75	0.045–0.055	0.051–0.092	0.055–0.072	0.059–0.069	
Min–max	0.043–0.343	0.041–0.119	0.047–0.093	0.049–0.087	

Abbreviation: s.d., standard deviation.

The differences in SD volume change at T4 versus T0 were statistically significant between the PLLA and control groups (−1.96 ± 1.53 vs. −0.02 ± 0.04, *p* = 0.002) and between the PLLA +1565‐nm NAFL and control groups (−1.48 ± 1.35 vs. −0.02 ± 0.04, *p* = 0.023). The decreases in SD area of influence in the PLLA +1565‐nm NAFL (−44.36 ± 36.44) and PLLA (−49.43 ± 25.16) groups were significantly greater than that in the 1565‐nm NAFL group (−14.30 ± 20.49, *p* = 0.044 and 0.014) and control group (−1.24 ± 2.89, *p* = 0.002 and 0.001), respectively. The changes in SD maximum depth before and after treatment were not significantly different among all four groups (Table 2).

**TABLE 2:** Comparison of SD volumes before and after treatment in the four groups.

	PLLA +1565‐nm NAFL group (*n* = 10)	PLLA group (*n* = 10)	1565‐nm NAFL group (*n* = 10)	Control group (*n* = 10)	*F*	*p*
SD volume (mean ± s.d.) (mm^3^)						
T0	2.37 ± 2.32	3.00 ± 1.89	2.86 ± 1.60	3.11 ± 1.46	0.31	0.89
T4	1.12 ± 0.73	1.51 ± 0.94	2.13 ± 0.58	2.93 ± 1.32	0.42	0.82
Difference between T0 and T4	−1.48 ± 1.35	−1.96 ± 1.53	−0.70 ± 0.67	−0.02 ± 0.04	6.29	0.00*
SD area of influence (mean ± s.d.) (mm^3^)						
T0	159.80 ± 32.87	160.90 ± 37.29	155.82 ± 31.60	158.38 ± 38.46	0.51	0.89
T4	73.82 ± 49.73	99.51 ± 56.54	99.03 ± 51.58	104.66 ± 44.32	0.75	0.53
Difference between T0 and T4	−44.36 ± 36.44	−49.43 ± 25.16	−14.30 ± 20.49	−1.24 ± 2.89	9.09	0.00*
SD maximum depth (mean ± s.d.) (mm)						
T0	0.08 ± 0.09	0.07 ± 0.03	0.06 ± 0.01	0.06 ± 0.01	—	—
T4	0.07 ± 0.06	0.06 ± 0.02	0.05 ± 0.09	0.06 ± 0.01	0.25	0.86
Difference between T0 and T4	−0.01 ± 0.01	−0.04 ± 0.09	−0.03 ± 0.08	−0.00 ± 0.00	0.68	0.57

Abbreviation: s.d., standard deviation.

* *p* < 0.05, indicating that the difference between a two‐by‐two comparisons was statistically significant.

The scores assigned by the blinded investigators at T4 are listed in Table 3. Evident clinical improvements in all treatment groups were observed. The SD area, SD degree of unevenness, and skin elasticity showed excellent improvements (> 75%) after treatment in the PLLA + 1565‐nm NAFL group. The percentages of subjects who had marked improvement in the SD area, the SD degree of unevenness, and the elasticity after treatment in the PLLA and 1565‐nm NAFL groups were 90.00% (9/10) and 30.00% (3/10), 90.00% (9/10) and 20.00% (2/10), and 70.00% (7/10) and 20.00% (2/10), respectively.

**TABLE 3:** Scoring (using a 4‐point grading scale) for area, degree of unevenness, elasticity, color, and overall score for the four groups.

	PLLA +1565‐nm NAFL group (*n* = 10)	PLLA group (*n* = 10)	1565‐nm NAFL group (*n* = 10)	Control group (*n* = 10)	*p*
Area					
0	0 (0.00%)	0 (0.00%)	1 (10.00%)	10 (100.00%)	0.000
1	1 (10.00%)	1 (10.00%)	6 (60.00%)	0 (0.00%)	
2	6 (60.00%)	9 (90.00%)	3 (30.00%)	0 (0.00%)	
3	3 (30.00%)	0 (0.00%)	0 (0.00%)	0 (0.00%)	
Degree of unevenness					
0	0 (0.00%)	0 (0.00%)	1 (10.00%)	10 (100.00%)	0.000
1	2 (20.00%)	1 (10.00%)	7 (70.00%)	0 (0.00%)	
2	5 (50.00%)	9 (90.00%)	2 (20.00%)	0 (0.00%)	
3	3 (30.00%)	0 (0.00%)	0 (0.00%)	0 (0.00%)	
Elasticity					
0	0 (0.00%)	0 (0.00%)	1 (10.00%)	10 (100.00%)	0.000
1	1 (10.00%)	3 (30.00%)	7 (70.00%)	0 (0.00%)	
2	6 (60.00%)	7 (70.00%)	2 (20.00%)	0 (0.00%)	
3	3 (30.00%)	0 (0.00%)	0 (0.00%)	0 (0.00%)	
Color					
0	9 (90.00%)	9 (90.00%)	9 (90.00%)	10 (100.00%)	0.788
2	1 (10.00%)	1 (10.00%)	1 (10.00%)	0 (0.00%)	
Overall scores					
Mean ± s.d.	6.70 ± 2.21	5.70 ± 1.25	3.60 ± 2.12	0.00 ± 0.00	0.000
Median	6	6	3	0	
*P*25–*P*75	5–9	5–6	3–4	0–0	
Min–max	4–11	3–8	0–8	0–0	

Abbreviation: s.d., standard deviation.

**FIGURE 1:**
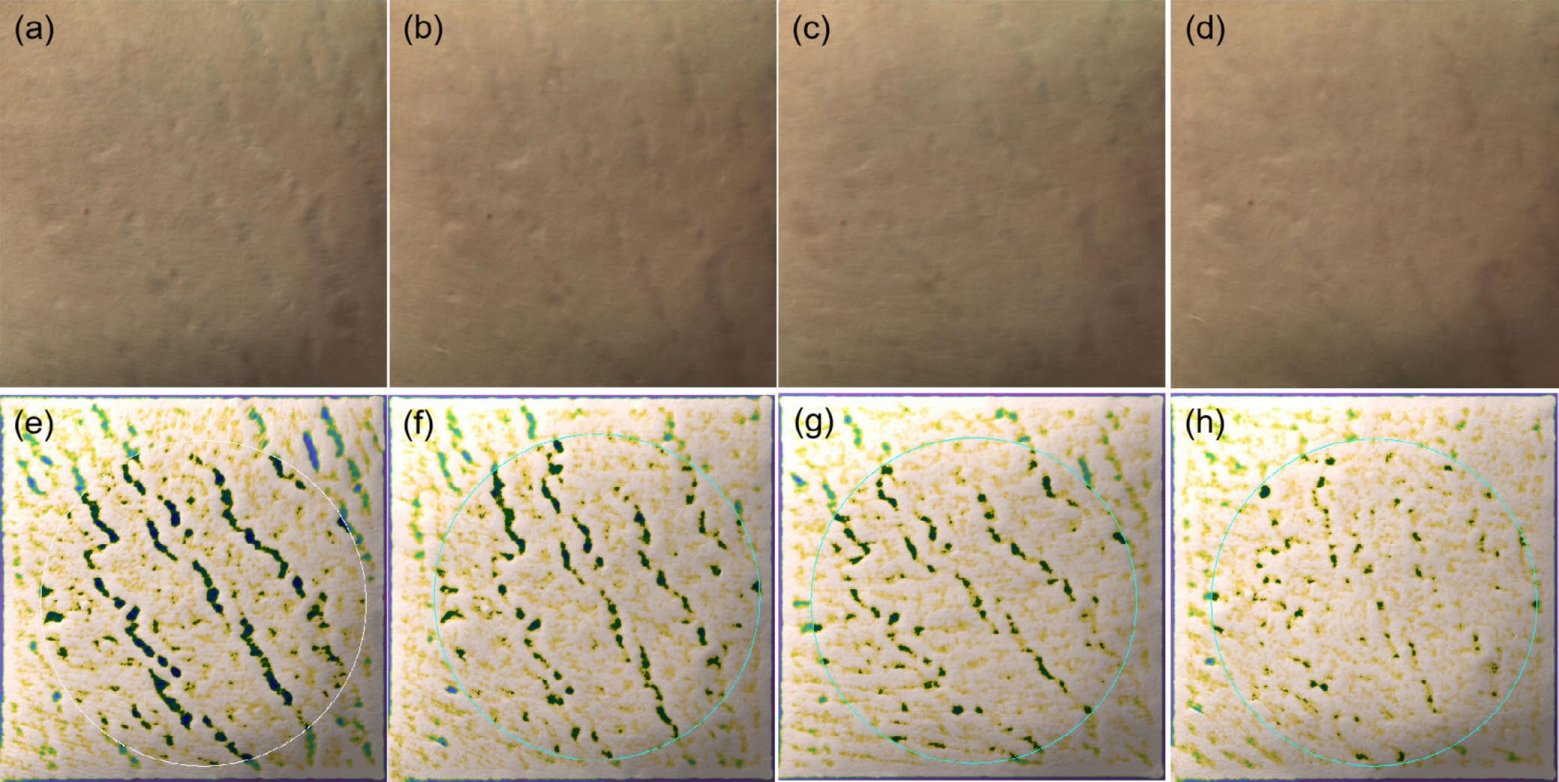
The evaluation of Antera 3D images showing the width of SD is decreased after each treatment compared to baseline. (a, e) Baseline, (b, f) 1 month after the first treatment of PLLA injection + 1565‐nm NAFL treatment, (c, g) 1 month after the second treatment of PLLA injection + 1565‐nm NAFL treatment, (d, h) 1 month after the third treatment of PLLA injection + 1565‐nm NAFL treatment.

**FIGURE 2:**
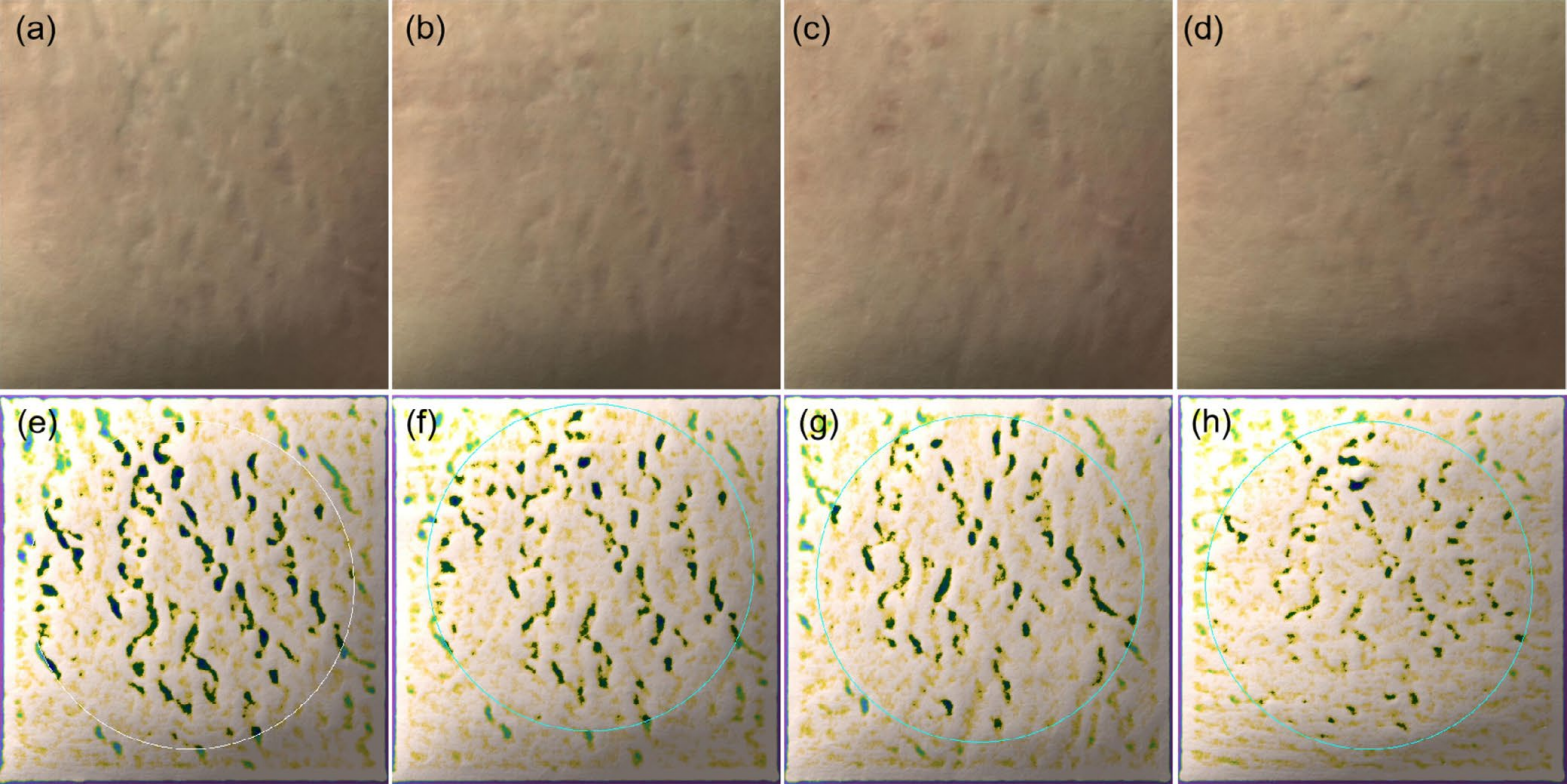
The evaluation of Antera 3D images showing the width of SD is decreased after each treatment compared to baseline. (a, e) Baseline, (b, f) 1 month after the first treatment of PLLA injection treatment, (c, g) 1 month after the second treatment of PLLA injection treatment, (d, h)1 month after the third treatment of PLLA injection treatment.

**FIGURE 3:**
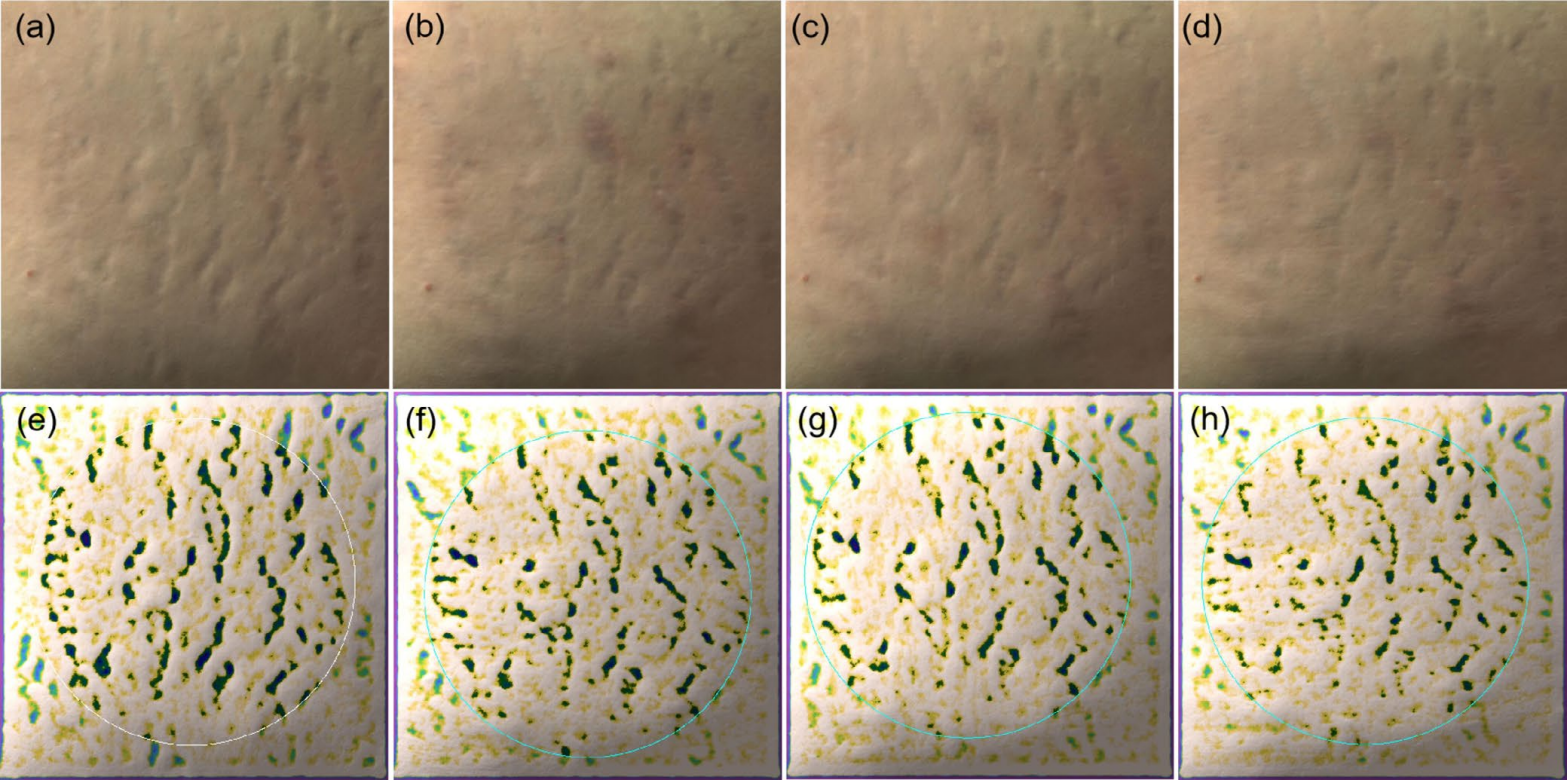
The evaluation of Antera 3D images showing the width of SD is decreased after each treatment compared to baseline. (a, e) Baseline, (b, f) 1 month after the first treatment of 1565‐nm NAFL treatment, (c, g) 1 month after the second treatment of 1565‐nm NAFL treatment, (d, h)1 month after the third treatment of 1565‐nm NAFL treatment.

**FIGURE 4:**
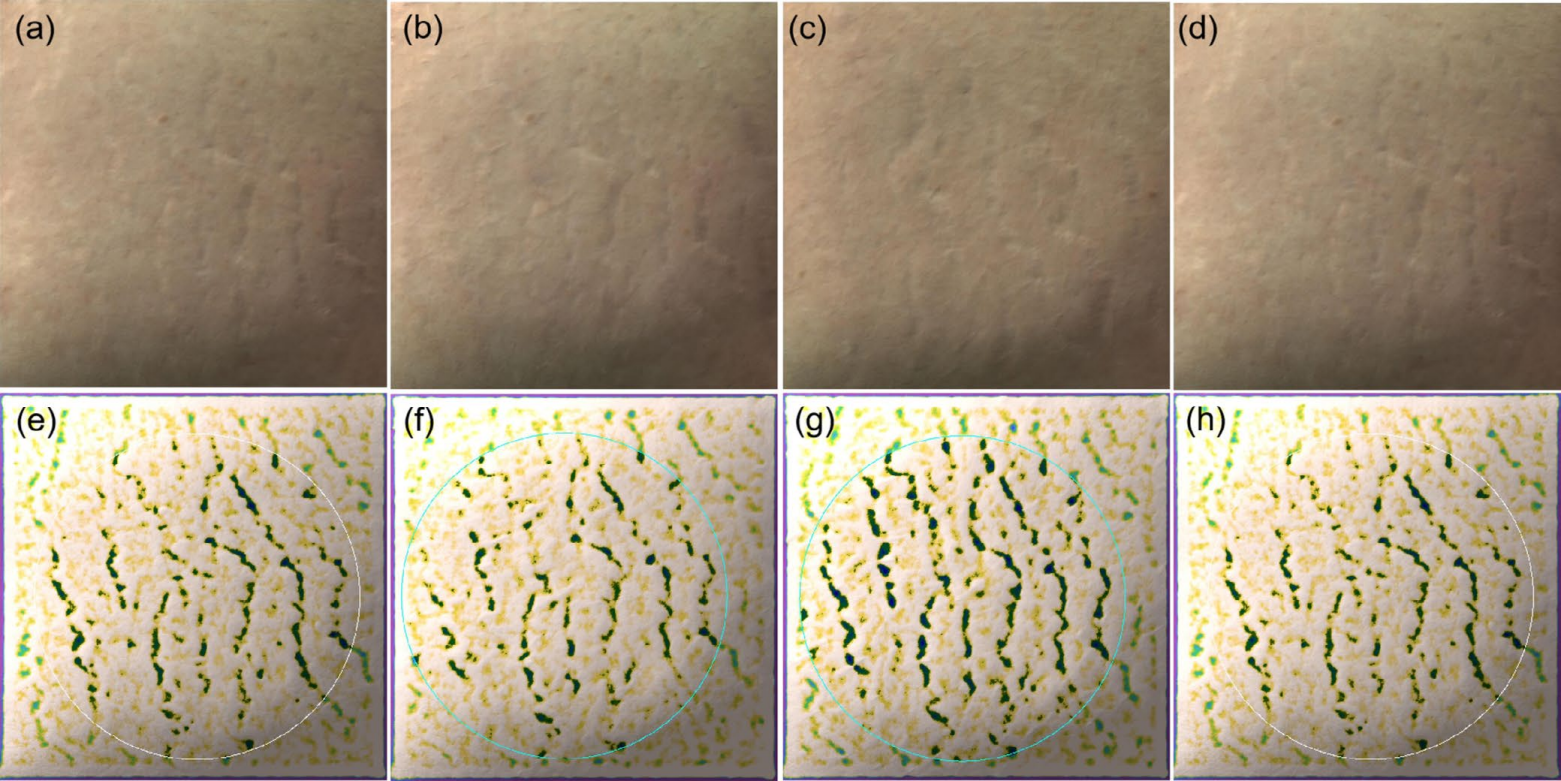
A photomicrograph of a section from SD 1 month after the last treatment with PLLA injection, (a, H&E × 400) Scattered distribution of PLLA particles between collagen fibers without inflammatory reaction, (b, H&E × 400 polariscope) PLLA particles have refractive properties, (c, picrosirius red × 400) thick intermingling bundles in the deep dermis with longitudinal sections and transverse ones, (d, picrosirius red × 400 polariscope) PLLA particles have refractive properties.

The overall efficacy scores for the PLLA, 1565‐nm NAFL, and PLLA +1565‐nm NAFL groups were 5.70 ± 1.25, 3.60 ± 2.12, and 6.70 ± 2.21, respectively (Table 3). The overall scores of efficacy evaluation of subjects in different subgroups were compared two by two in Table 4. The PLLA, 1565‐nm NAFL, and PLLA +1565‐nm NAFL groups all had statistically significant differences compared with the control group (95% confidence intervals [CIs] = 3.63–7.77, 1.52–5.67, and 4.63–8.77, respectively). The overall efficacy scores for subjects in the PLLA +1565‐nm NAFL and PLLA groups were higher than those in the 1565‐nm NAFL group, and the differences were statistically significant (95% CIs = 1.03–5.17 and 0.03–4.17, respectively). There were no significant differences in all efficacy parameters and indexes between the PLLA +1565‐nm NAFL and PLLA groups. Figures 1, 2, 3 illustrate the analysis of Antera 3D images for PLLA injection combined with 1565‐nm NAFL treatment, PLLA injection alone, and 1565‐nm NAFL treatment alone, revealing a reduction in SD width after each treatment when compared to baseline measurements. Figure 4 presents images of participants from the control group.

**TABLE 4:** Results of two‐by‐two comparisons of total scores of efficacy evaluation for the four groups.

Groups	Difference in means	95% CI		Statistical significance
PLLA +1565‐nm NAFL group vs. PLLA group	1.0000	−1.0664	3.0664	—
PLLA +1565‐nm NAFL group vs. 1565‐nm NAFL group	3.1000	1.0336	5.1664	***
PLLA +1565‐nm NAFL group vs. control group	6.7000	4.6336	8.7664	***
PLLA group vs. PLLA +1565‐nm NAFL group	−1.0000	−3.0664	1.0664	—
PLLA group vs. 1565‐nm NAFL group	2.1000	0.0336	4.1664	***
PLLA group vs. control group	5.7000	3.6336	7.7664	***
1565‐nm NAFL group vs. PLLA +1565‐nm NAFL group	−3.1000	−5.1664	−1.0336	***
1565‐nm NAFL group vs. 1565‐PLLA group	−2.1000	−4.1664	−0.0336	***
1565‐nm NAFL group vs. control group	3.6000	1.5336	5.6664	***
Control group vs. PLLA +1565‐nm NAFL group	−6.7000	−8.7664	−4.6336	***
Control group vs. PLLA group	−5.7000	−7.7664	−3.6336	***
Control group vs. 1565‐nm NAFL group	−3.6000	−5.6664	−1.5336	***

*** *p* < 0.001, indicating a statistically significant difference for a two‐by‐two comparison.

### 3.2 PLLA Injections Stimulate the Regeneration of Collagen Type III and Decrease the Ratio of Type I to Type III Collagens

Tissue histology studies revealed the fragmentation and fraying of collagenous and elastic fibers in all sections at T0. There were more abundant and organized collagen fibers and thickened cuticles after treatment with the 1565‐nm NAFL, PLLA, and PLLA +1565‐nm NAFL compared to the control group (Table 5). At T4, the PD values for type I collagen fibers were measured as follows: 18.58 ± 4.39 for the 1565‐nm NAFL group, 17.01 ± 3.16 for the PLLA group, 16.29 ± 4.52 for the PLLA +1565‐nm NAFL group, and 21.32 ± 2.86 for the control group. There were no differences in PD values of type I collagen fibers in the 1565‐nm NAFL, PLLA, and PLLA +1565‐nm NAFL groups at T4 compared to the control group (*p* = 0.301, *p* = 0.462, and *p* = 0.089, respectively). At T4, the PD values for type III collagen fibers were measured as follows: 10.38 ± 2.41 for the 1565‐nm NAFL group, 11.02 ± 2.48 for the PLLA group, 11.73 ± 1.97 for the PLLA +1565‐nm NAFL group, and 6.92 ± 0.85 for the control group. The PD values of type III collagen fibers were significantly higher at T4 for the 1565‐nm NAFL, PLLA, and PLLA +1565‐nm NAFL groups (*p* = 0.032, *p* = 0.002, and *p* = 0.016, respectively) compared to the control group. Type III collagen PD values were significantly higher in the PLLA +1565‐nm NAFL than the 1565‐nm NAFL group at T4 (*p* = 0.041). At T4, the ratio of type I to type III collagens was measured as follows: 2.68 ± 1.01 for the 1565‐nm NAFL group, 1.57 ± 0.84 for the PLLA group, 1.19 ± 0.79 for the PLLA +1565‐nm NAFL group, and 3.31 ± 1.22 for the control group. The ratio of type I to type III collagens decreased in the PLLA +1565‐nm NAFL and PLLA groups at T4 compared to the control group (*p* = 0.011 and *p* = 0.028, respectively); there was no difference observed in the 1565‐nm NAFL group at T4 as compared with the control group (*p* = 0.323). At T4, average OD values of elastic fibers (Masson) were measured as follows: 30701.33 ± 2571.34 for the 1565‐nm NAFL group, 31709.91 ± 3022.38 for the PLLA group, 33134.15 ± 5371.65 for the PLLA +1565‐nm NAFL group, and 27315.84 ± 2291.01 for the control group. In the PLLA +1565‐nm NAFL, 1565‐nm NAFL, and PLLA groups, the average OD values of elastic fibers were significantly higher at T4 than the control group (*p* = 0.001, *p* = 0.012, and *p* = 0.004, respectively). Figures S2–S5 present pathological images of abdominal skin tissue under different conditions.

**TABLE 5:** Types I and III collagen and elastic fibers OD in the four treatment groups.

Histopathology features	PLLA +1565‐nm NAFL group (*n* = 10)	PLLA group (*n* = 10)	1565‐nm NAFL group (*n* = 10)	Control group (*n* = 10)
Type I collagen PD (%) at T0	20.98 ± 2.37	21.36 ± 2.19	20.87 ± 2.05	21.22 ± 3.53
Type I collagen PD (%) at T4	16.29 ± 4.52	17.01 ± 3.16	18.58 ± 4.39	21.32 ± 2.86
Type III collagen PD (%) at T0	6.59 ± 1.25	7.01 ± 3.28	6.49 ± 2.37	6.83 ± 1.74
Type III collagen PD (%) at T4	11.73 ± 1.97	11.02 ± 2.48	10.38 ± 2.41	6.92 ± 0.85
Type I:type III collagen ratio at T0	3.17 ± 0.75	2.98 ± 0.92	3.31 ± 1.09	3.25 ± 0.97
Type I: type III collagen ratio at T4	1.19 ± 0.79	1.57 ± 0.84	2.68 ± 1.01	3.31 ± 1.22
Elastic fibers PD (%) at T0	1.78 ± 0.56	1.06 ± 0.93	1.53 ± 0.76	1.12 ± 0.35
Type I:type III collagen ratio at T4	5.87 ± 2.19	5.32 ± 1.21	2.63 ± 0.47	1.07 ± 0.41
Elastic fibers OD (Masson) at T0	29142.45 ± 4251.84	30193.43 ± 4259.10	29415.57 ± 3237.74	28381.01 ± 2953.12
Elastic fibers OD (Masson) at T4	33134.15 ± 5371.65	31709.91 ± 3022.38	30701.33 ± 2571.34	27315.84 ± 2291.01

**FIGURE 5:**
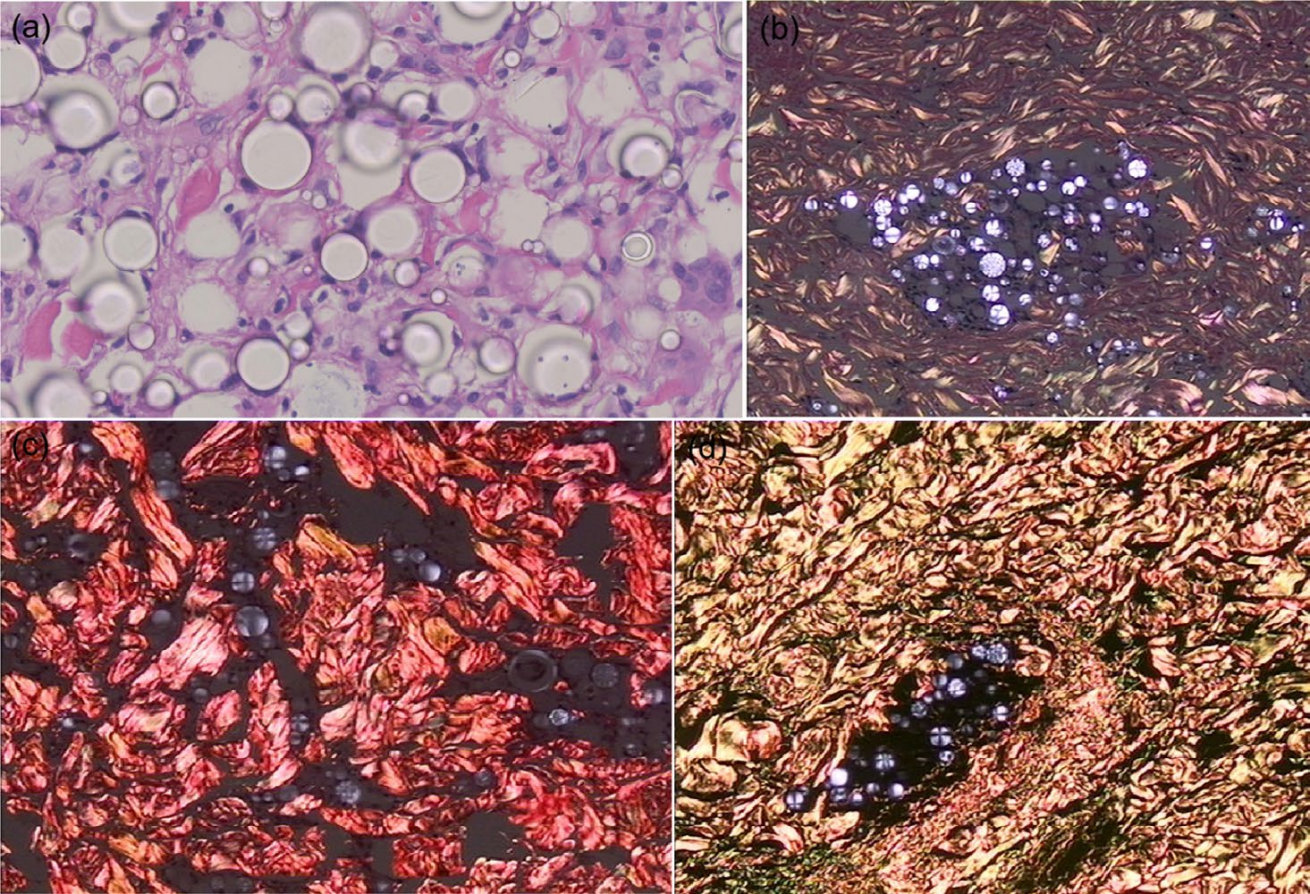
A photomicrograph of a section from SD 1 month after the last treatment with PLLA injection, (a, H&E x 400) Scattered distribution of PLLA particles between collagen fibers without evident inflammatory reaction, (b, H&E x 400 polariscope) PLLA particles have refractive properties, (c, picrosirius red x 400) thick intermingling bundles in the deep dermis with longitudinal sections and transverse ones, (d, picrosirius red x 400 polariscope) PLLA particles have refractive properties.

PLLA particles were evenly dispersed at the injection sites without signs of inflammation in the PLLA group at T4. No adverse reactions (e.g., nodule formation) were found in the injection areas. In addition, the PLLA particles were found in full contact with cells without compression of tissue deformation (Figure 5).

### 3.3 No Hyperpigmentation Was Observed in the PLLA +1565‐Nm NAFL Group

Patients experienced fleeting burning sensations and pain after treatments, with temporary erythema and wheals that resolved within 24 h. Slight pigmentation was observed in four patients in the 1565‐nm NAFL group. No hyperpigmentation was observed in the PLLA + 1565‐nm NAFL group. The percentages of patients who experienced significant or intolerable pain during 1565‐nm NAFL, PLLA, or PLLA + 1565‐nm NAFL were 40.0% (5/10), 50.0% (5/10), and 60.0% (6/10), respectively. No long‐lasting or severe adverse effects were associated with any of these treatments. A total of 80.00% (8/10), 60.00% (6/10), and 30.00% (3/10) of the subjects were very satisfied or satisfied with the PLLA + 1565‐nm NAFL, PLLA, and 1565‐nm NAFL treatments, respectively.

## 4 Discussion

Although several effective therapies for SD have been reported, there is no widely accepted modality or effective therapy for the management of SD. Among all therapies, non‐ablative fractional lasers and filler injections are more commonly used due to their wide safety margin and patient tolerance. In this study, we found that PLLA is a safe and efficacious option for treating SD, showing better outcomes than 1565‐nm NAFL. In addition, PLLA could stimulate collagen regeneration in SD without an obvious inflammatory response.

PLLA is a synthetic, biocompatible, biodegradable, absorbable, and biostimulatory polymer that provides soft tissue augmentation. It can be injected into the reticular dermis or subcutaneous layers to stimulate collagenous formation throughout several treatments, leading to gradual volume restoration and correction/restoration of facial volume loss associated with aging [15]. Composite microparticles usually trigger a foreign body inflammatory response, which is followed by encapsulation of the microparticles, fibroplasia, and resultant type I collagen deposition in the extracellular matrix. In addition to the immediate volume‐filling effect [16], PLLA can induce long‐term effects by promoting collagen synthesis by fibroblasts even after PLLA microparticle degradation [17]. In a previous study, PLLA showed stronger collagenous stimulation, but less inflammation, as compared with other dermal fillers such as poly‐D‐lactic acid (PDLA) and meso‐PLA (PDLLA). Furthermore, PLLA could contain collagen and other substances beneficial to skin repair and collagen regeneration that can be released slowly through PLLA degradation to achieve better cosmetic effects [18]. We found that PLLA injections improved skin elasticity and reduced SD area. The mechanism of action by which PLLA increases collagen synthesis follows the principle of immunological response and a subclinical foreign body reaction [19]. It is suggested that PLLA particles can be encapsulated and surrounded by mast cells, mononuclear macrophages, and lymphocytes 3 weeks after the injection. Continuous collagenesis occurs, and the synthesized collagen fibers surround the PLLA particles [10]. It is important to explore why PLLA injection alone might outperform the combination of PLLA injection and laser treatment. The interaction between PLLA injections and laser treatments requires careful consideration due to the potential impact of laser energy on PLLA's effectiveness. PLLA works by gradually breaking down into lactic acid, which stimulates collagen production over time. However, the heat generated by laser treatments could interfere with this process by accelerating the degradation of PLLA particles. This premature breakdown may reduce the longevity of their collagen‐stimulating effects, as the particles might not have sufficient time to induce optimal collagen synthesis. The timing and sequence of these procedures are also critical—performing laser treatments too soon after PLLA injections could prematurely degrade the particles before they have fully integrated into the tissue. This could explain why PLLA injections alone might outperform the combination of PLLA and laser treatments. To minimize these risks, it is recommended to carefully space out the treatments, allowing adequate time for PLLA to take effect before introducing laser energy, or to use lower‐energy laser settings or alternative non‐heat‐based devices. Ultimately, optimizing the timing, sequence, and energy settings of these treatments is essential for achieving the best outcomes, and further scientific investigation is warranted to confirm this hypothesis.

Histologically, SD and scar healing are very similar. Ablative fractional lasers can treat atrophic scarring by dermal remodeling. Recently, researchers have attempted to use them to treat SD. Non‐ablative fractional lasers can cause thermal damage to the skin and increase the amount of collagen and elastic fibers in treated lesions [20]. In addition, the study suggests that optimal laser treatment parameters are 30–50 mJ/μb, and the researchers posit that the stamping mode energy pattern is the best for SD treatment, ensuring the effectiveness and consistent distribution of microbeams into the skin. The superiority of the treatment effect lies more in the length, width, and density of striae compared to the maturity of SD. We found that 1565‐nm NAFL treatment improved skin elasticity and color and reduced the SD degree of unevenness.

The present study demonstrated that PLLA injections and PLLA +1565‐nm NAFL stimulated the regeneration of collagen type III and decreased the ratio of type I to type III collagens. The histopathological abnormalities of striae gravidarum are mainly found in collagens, elastic fibers, and fibrils [21]. Striae gravidarum promote fibroblast activity, leading to increased protein catabolism and alterations to collagen and elastic fibers. During the early stage of SD, macrophages and mast cells accumulate around dermal elastic fibers, causing them to dissolve and lose their functions; in addition, horizontal eosinophilic collagen bundles appear in the shallow layer of the dermis. This is accompanied by shrinkage of the epidermal layers and the disappearance of stripe edges [22]. The type I to type III collagen ratio of normal skin is approximately 1.5, but this ratio increases to 3–5.8 in scars, according to a previous report [23]. We found that the PLLA injections used in this study induced collagen regeneration in a noninflammatory way. Biologic responses to PLLA implants are histologically characterized by a classic foreign body granuloma. Three months after subcutaneous injection, PLLA microspheres are surrounded by macrophages, lymphocytes, giant cells, and a fibrous tissue capsule consisting mostly of collagen. Six months after injection, the microparticles are porous and deformed. At 9 months, there is no evidence of residual polymers or surrounding fibrosis [24]. We acquired biopsy specimens 1 month after the last injection, and there was no obvious inflammation in our cohort. This outcome may be attributed to the timing and location of the sampling, or perhaps other reasons. Advancements in the formulation and processing of PLLA have significantly enhanced its biocompatibility, thereby reducing or even eliminating the typical foreign body reaction. These improvements may include surface coatings or treatments applied to PLLA materials, which are designed to minimize the immune response. Additionally, the degradation rate of PLLA plays a crucial role in its interaction with the body. A slower degradation rate allows the material to break down gradually, preventing an overwhelming release of degradation products into the local environment and thus mitigating the potential for a significant immune response. It is crucial to consider these factors when interpreting the results of histological evaluations of PLLA and to conduct further studies to confirm these observations under varied conditions.

## 5 Conclusion

The present comparative study demonstrated that PLLA injection, 1565‐nm NAFL, and the combined treatment showed their efficacies against SD and had no long‐term adverse effects. Both PLLA injections and the PLLA injection combined with 1565‐nm NAFL showed better outcomes than the 1565‐nm NAFL only. Collagenic and elastogenic neoformation were observed after PLLA injections, with no inflammatory reaction in the subcutaneous tissue. The PLLA injection is a promising treatment for SD.

### 5.1 Limitations

The present study included a limited number of patients, did not include patients with dark‐skin types, and had a limited follow‐up period. Other self‐control studies involving larger samples are necessary to corroborate our findings.

## Author Contributions

**Huanhuan Qu:** writing – original draft. **Huanhuan Qu:** data curation. **Li Wang:** data curation. **Wuyan Xin:** investigation. **Meiheng Lu:** investigation. **Huan Jing:** investigation. **Lin Gao:** conceptualization, methodology. **Gang Wang:** supervision, writing – review and editing.

## Ethics Statement

The authors confirm that the ethical policies of the journal, as noted on the journal's author guidelines page, have been adhered to and the appropriate ethical review committee approval has been received. The study was reviewed and approved by the ethical committee of Xijing Hospital, Air Force Medical University (No. KY20232083) on 21st March 2023. Written informed consent was obtained from all patients. The patients in this manuscript have given written informed consent to the publication of their case details.

## Conflicts of Interest

The authors declare no conflicts of interest.

## Supporting information


Figures S1–S5.

## Data Availability

All data supporting the findings of this study are available.
