# Supplementary material for: Clinical Efficacy Comparisons Between Poly‐L‐Lactic Acid Injections and Non‐Ablative 1565‐nm Fractional Laser for Treatment of Striae Distensae—A Randomized Trial
Source: J Cosmet Dermatol. 2025 Jul 15;24(7):e70338. doi: 10.1111/jocd.70338 (PMC12261269; doi:10.1111/jocd.70338)
Supplement: Supplementary file 1 — Figures S1–S5. [file JOCD-24-e70338-s001.docx]

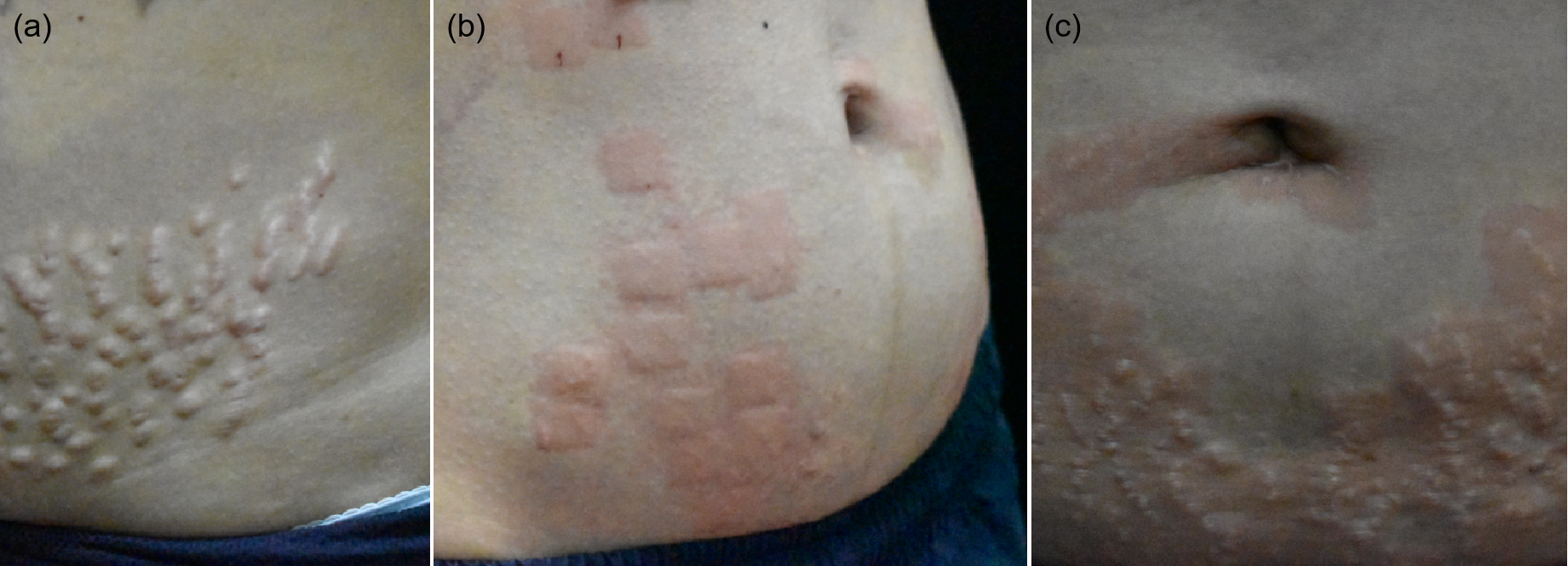


Supplementary Fig.1. Immediate reactions after treatments. (a) local swelling after the PLLA injection treatment, (b) local erythema and urticaria-like changes after 1565-nm NAFL treatment, (c) combined treatment responses of (a) and (b) after PLLA injection + 1565-nm NAFL treatment


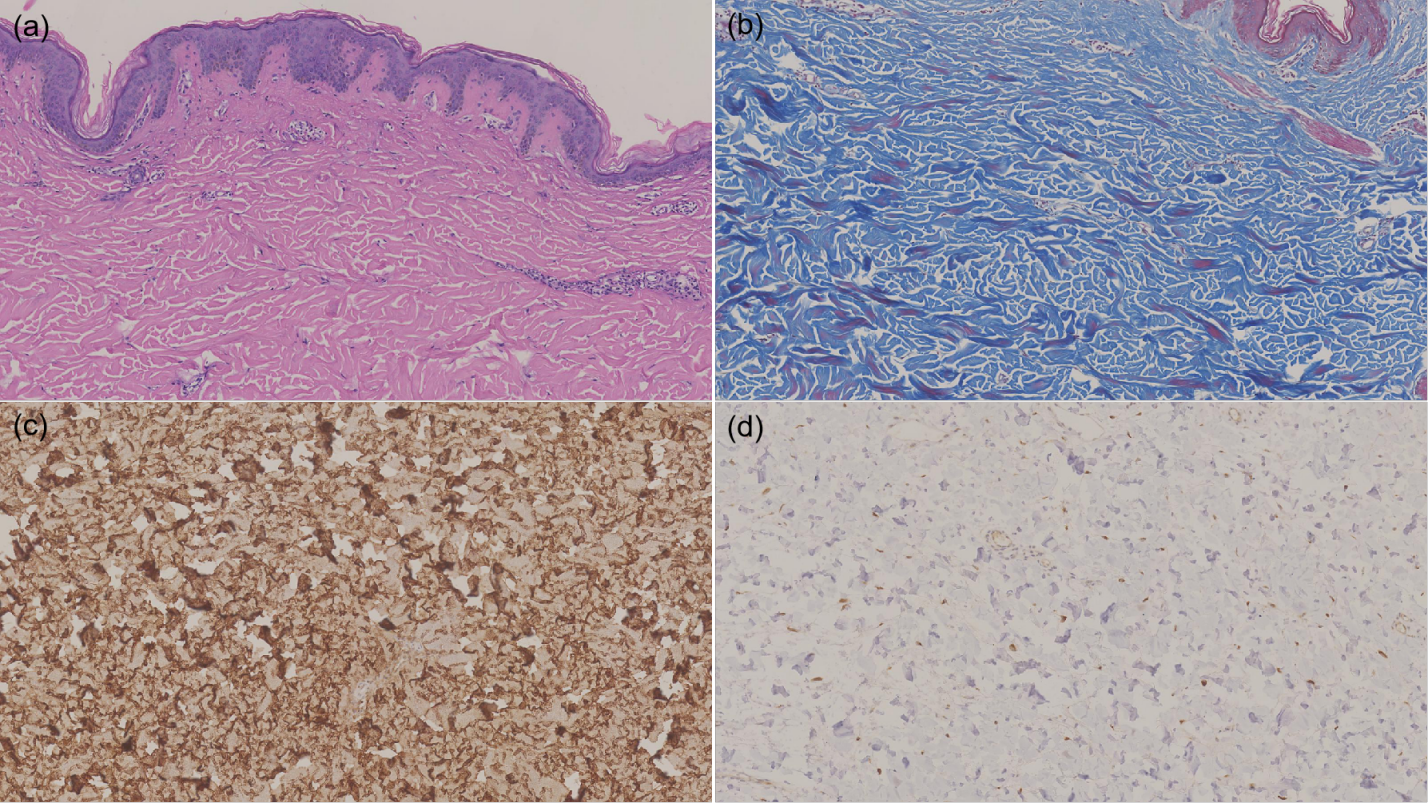


Supplementary Fig.2. (a H&E x 125), (b, Masson trichrome x 250), (c, Collagen I x 400) and (d, Collagen III x 400) Pathological images of abdominal skin tissue of normal control non-SD patients


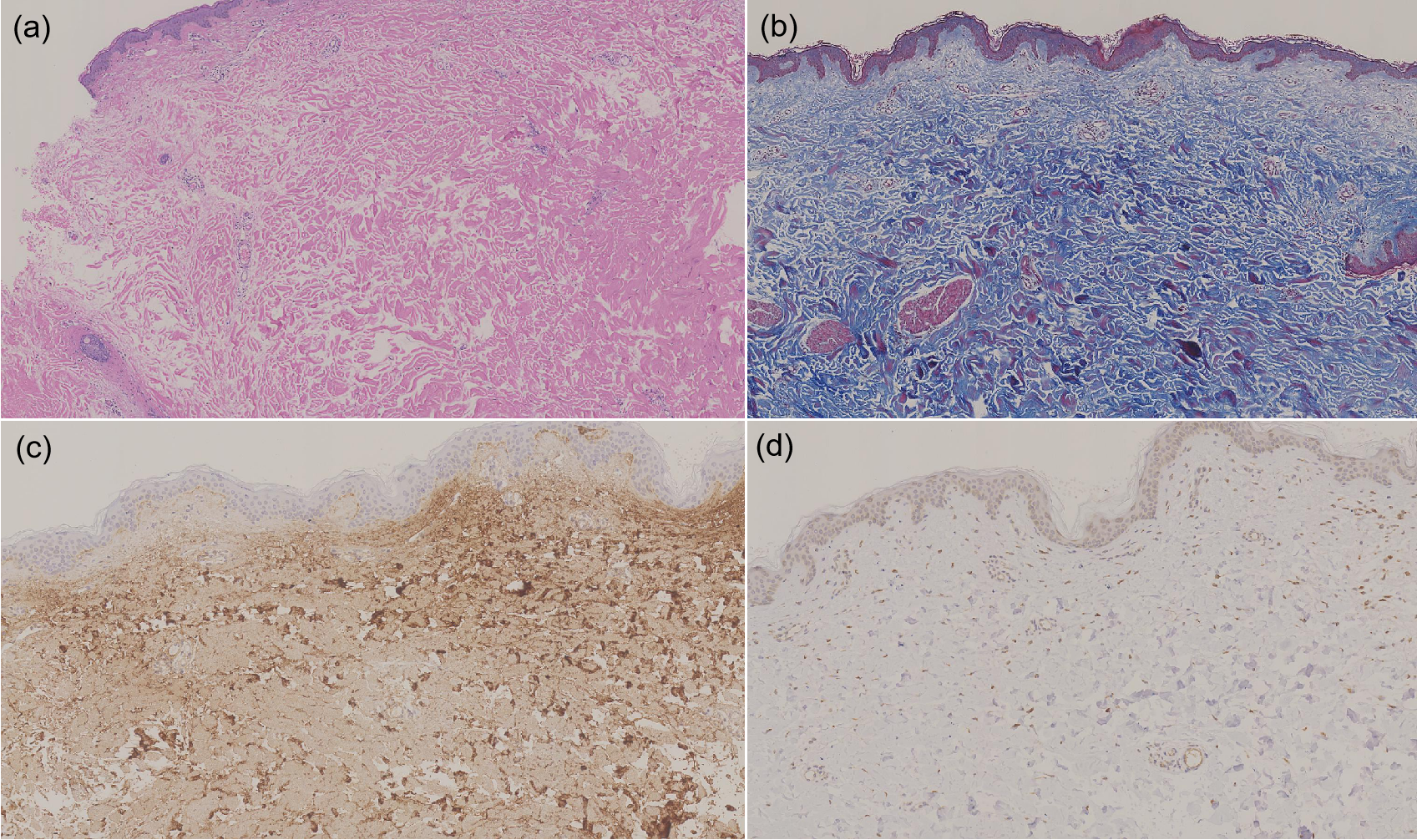


Supplementary Fig.3. (a H&E x 125), (b, Masson trichrome x 250), (c, Collagen I x 400) and (d, Collagen III x 400) Pathological images of abdominal skin tissue of SD without treatment in control group


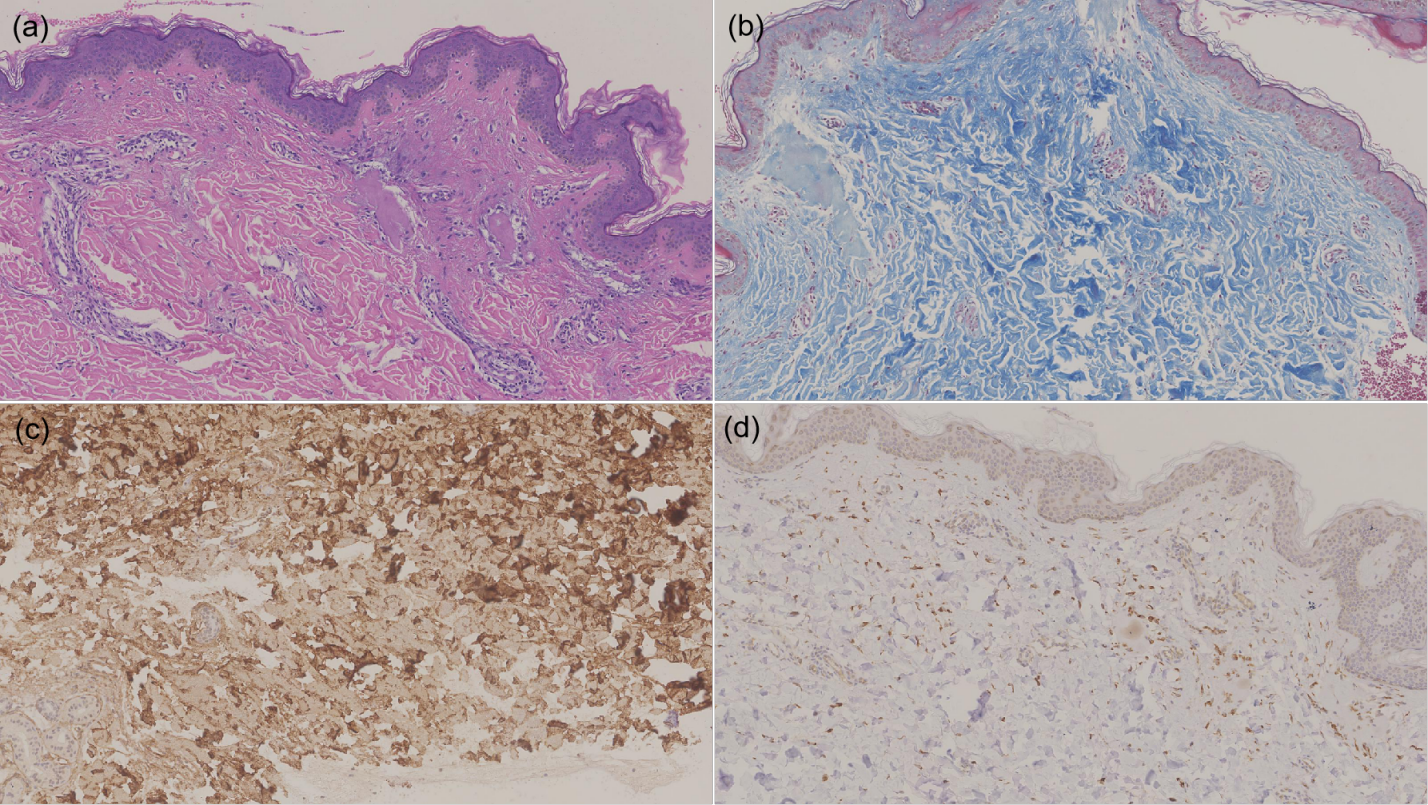


Supplementary Fig.4. (a H&E x 125), (b, Masson trichrome x 250), (c, Collagen I x 400) and (d, Collagen III x 400) Pathological images of abdominal skin tissue of SD after 1565-nm NAFL treatment, it shows that abundant collagen as thin fibers within the papillary dermis


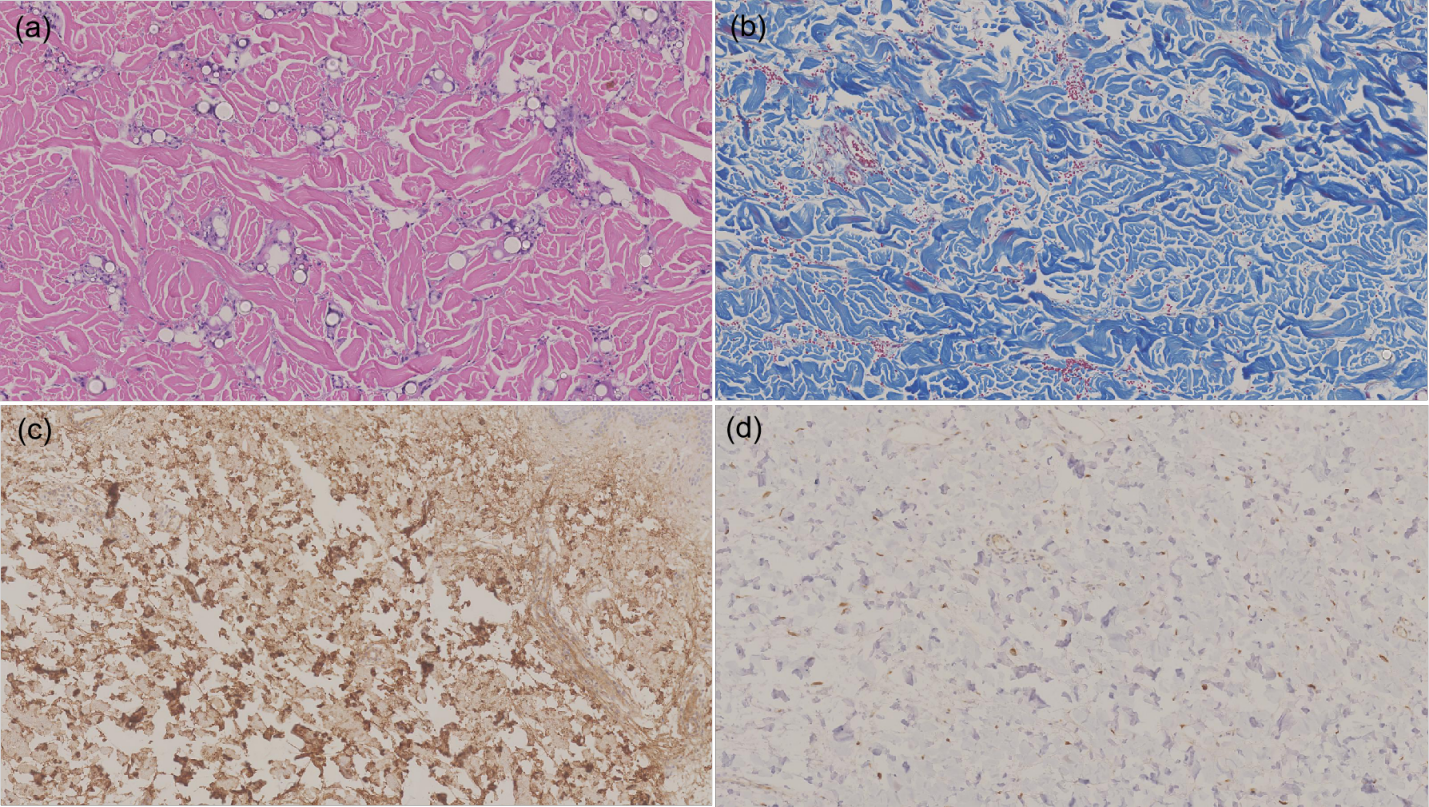


Supplementary Fig.5. (a H&E x 125), (b, Masson trichrome x 250), (c, Collagen I x 400) and (d, Collagen III x 400) Pathological images of abdominal skin tissue of SD after PLLA injection treatment, abundant collagen as thin fibers within the papillary dermis and thick bundles in the deeper was seen, and it also shows scattered PLLA particles between collagen fibers without inflammatory reaction
